# The Impact and Response to Climate Change as Lived by Older Adults

**DOI:** 10.1093/geroni/igab046.2258

**Published:** 2021-12-17

**Authors:** Robin Fenley, Toni Antonucci, Robin Fenley

## Abstract

A growing body of literature documents the domino effects of climate change on the planet and all life. Chief among these changes is the rise in global temperatures, triggering record numbers of heatwaves, and stronger, more dangerous hydrologic events. While climate change looms as a preeminent threat to our planet and future, the public health and human rights ramifications are already apparent. As with many issues in this realm, the effects are felt to a greater degree by our aging populations. As disasters grow in frequency, the more vulnerable populations are at greater risk for more serious outcomes – and will suffer disproportionately from the effects of climate change – resulting in greater inequalities. With the consequences of climate change growing more drastic, these outcomes can be expected to climb unless sufficient measures are enacted to combat global warming. In this symposium we will highlight the link between climate change and its impact on the human rights of older adults, and how climate change threatens progress across the Sustainable Development Goals (SDG) - a blueprint for a more equitable and healthier planet - if decisive actions are not taken. This symposium will demonstrate what valuable opportunities exist to accelerate progress by leveraging the links between SDGs to combat inequalities and climate change. Panelists will discuss the adverse effects of climate change, the human rights and psychological impacts on older adults, and potential action steps and strategies for older persons to become empowered as advocates for climate change reform.

